# Dating and localizing an invasion from post-introduction data and a coupled reaction–diffusion–absorption model

**DOI:** 10.1007/s00285-019-01376-x

**Published:** 2019-05-16

**Authors:** Candy Abboud, Olivier Bonnefon, Eric Parent, Samuel Soubeyrand

**Affiliations:** 1 BioSP, INRA, 84914 Avignon, France; 2 UMR 518 Math. Info. Appli., AgroParisTech, Paris, France; 3 UMR 518 Math. Info. Appli., INRA, Paris, France

**Keywords:** Partial differential equation, Reaction–diffusion, Diffusion–absorption, Bayesian inference, Mechanistic-statistical approach, Biological invasions, Disease dynamics, *Xylella fastidiosa*, 62F15, 65M06, 35K10

## Abstract

Invasion of new territories by alien organisms is of primary concern for environmental and health agencies and has been a core topic in mathematical modeling, in particular in the intents of reconstructing the past dynamics of the alien organisms and predicting their future spatial extents. Partial differential equations offer a rich and flexible modeling framework that has been applied to a large number of invasions. In this article, we are specifically interested in dating and localizing the introduction that led to an invasion using mathematical modeling, post-introduction data and an adequate statistical inference procedure. We adopt a mechanistic-statistical approach grounded on a coupled reaction–diffusion–absorption model representing the dynamics of an organism in an heterogeneous domain with respect to growth. Initial conditions (including the date and site of the introduction) and model parameters related to diffusion, reproduction and mortality are jointly estimated in the Bayesian framework by using an adaptive importance sampling algorithm. This framework is applied to the invasion of *Xylella fastidiosa*, a phytopathogenic bacterium detected in South Corsica in 2015, France.

**Electronic supplementary material:**

The online version of this article (10.1007/s00285-019-01376-x) contains supplementary material, which is available to authorized users.

## Introduction

Biological invasions have long been an important topic for biologists and mathematicians because of their impact on the environment, indigenous species, and health of humans, animals and plants (Andow et al. 1990, 1993; Baker 1991; Hengeveld 1989; Kermack and McKendrick 1927; Richardson and Bond 1991; Simberloff 1989; Anderson et al. 1996; Shigesada and Kawasaki 1997; Weinberger 1978). Biological invasions are generally viewed as the result of a process with four stages: arrival, establishment, spread and concentration (Reise et al. 2006; Vermeij 1996). Each stage of the invasion process has been a core topic in mathematical modeling since the mid-twentieth century (Fisher 1937; Mollison 1977; Okubo 1980; Shigesada et al. 1995; Skellam 1951), and better understanding processes governing invasions is chiefly relevant for improving surveillance and control strategies. In particular, extensive researches have been conducted in the intents of reconstructing the past dynamics (Boys et al. 2008; Roques et al. 2016; Soubeyrand and Roques 2014) of alien species and predicting their future spatial extents (Chapman et al. 2015; Peterson et al. 2003). In this context, partial differential equations offer a rich and flexible framework that has been applied to a large number of invasions (Gatenby and Gawlinski 1996; Lewis, MA and Kareiva, P 1993; Murray 2002; Okubo and Levin 2002; Turchin 1998). Even though a partial differential equation does not describe all the processes involved in an ecological dynamics, it can help in understanding its important properties and inferring its major components, such as dates and sites of invasive-species introductions.

Consider as an example the emergence of *Xylella fastidiosa* (Xf), a phytopathogenic bacterium detected in South Corsica, France, in 2015 and currently present in a large part of this island (Denancé et al. 2017b; Soubeyrand et al. 2018). This plant pathogen has the potential to cause a major sanitary crisis in France, typically like in Italy, where a large number of infected olive trees dried and died, causing serious damages to olive cultivation. To avoid such a situation, the French General Directorate of Food (DGAL) implemented enhanced control and surveillance measures after the first *in situ* detection of Xf in Corsica, which generated a data set consisting of a spatio-temporal point pattern (i.e. the locations and dates of plant samples) marked by a binary variable indicating the result of the diagnostic test (i.e. indicating if the plant sample is positive or negative to Xf).

In this example, only post-introduction data are available (i.e. data collected over a temporal window covering a period after the introduction time), and we precisely propose in this article an approach for estimating the date and the site of the introduction using such observational partial data. It has however to be noted that estimating the introduction point from post-introduction data requires the estimation of the propagation characteristics of the invasive species (and *vice versa*) because these characteristics link the introduction and the observations. Thus, in this paper, we aim at jointly estimating the date and site of the introduction, and other parameters related to growth, dispersal and death that govern the post-introduction dynamics.

Such a joint estimation was proposed by Soubeyrand and Roques (2014) with a simple reaction–diffusion model and was applied to simulated data. It was developed in a mechanistic-statistical framework that has often been used to describe and infer ecological processes. This framework combines a mechanistic model for the dynamics of interest, a probabilistic model for the observation process and a statistical procedure for estimating model parameters (Berliner 2003; Lanzarone et al. 2017; Roques et al. 2011; Soubeyrand et al. 2009a, b; Wikle 2003a, b). We adapted this framework for dating and localizing the introduction of an invasive species by taking into account spatial heterogeneities in growth and mortality. Precisely, we built a mechanistic model yielding the probability for the invasive species to occupy any spatial units at any time. This spatio-temporal function, with values in [0, 1], satisfies (i) a reaction–diffusion equation that describes the spread of the alien species in a sub-domain of the study domain and (ii) a diffusion–absorption equation that describes the dispersal and the death of the alien species in the complementary sub-domain. Typically, the partition into the two sub-domains can be determined by environmental variables affecting the growth and mortality of the invasive species (e.g. host/non-host environment, low/high winter temperature, and presence/absence of nutrients). In addition, our model assumes that there is only one introduction point (in time and space) that governs the emergence of the invasive species and that eventual other introduction points have negligible effects on the dynamics.

Estimation of model parameters, including the time and the location of the introduction, is carried out in the Bayesian framework with the adaptive multiple importance sampling algorithm (AMIS; Cornuet et al. 2012). Our main motivation for using AMIS is the gain in computation time with respect to Markov chain Monte Carlo (MCMC) often used in the mechanistic-statistical framework (see references above). From an example in population genetics, Cornuet et al. (2012) observed that AMIS was 6 times faster than MCMC for providing similar posteriors with a slightly better repeatability in the case of AMIS (without parallelization). The authors mentioned that AMIS is particularly interesting in cases where the likelihood is computationally expensive (like in our case) because all particles simulated during the process are recycled, which minimizes the numbers of calls of the likelihood function. In addition, like other adaptive importance sampling algorithms (Bugallo et al. 2015), AMIS can be easily parallelized and its tuning parameters are automatically adapted across the algorithm iterations.

In our framework, the two sub-domains, where the reaction–diffusion and diffusion–absorption equations are defined, are obtained by thresholding a spatial variable. The threshold value is determined with a selection criterion. Four criteria are considered: the Bayesian information criterion (BIC; Schwarz et al. 1978), two versions of the deviance information criteria (DIC; Gelman et al. 2003; Spiegelhalter et al. 2002) and a predictive information criterion (IC; Ando 2011). In the Xf case study, the two sub-domains are defined by thresholding the average of the minimum daily temperature in January and February, the two coldest months of the year in Corsica. Indeed, winter temperature has been inferred as an important environmental factor governing the dynamics of Xf and the level of disease severity caused by Xf (Costello et al. 2017; Feil et al. 2003; Feil and Purcell 2001; Henneberger 2003; Purcell 1977; Purcell et al. 1980). For instance, isolines for the average minimum daily temperature in January have been shown to be quite consistent with regions in the United States that are exposed to different levels of severity of the Pierce’s disease of grape caused by Xf (Anas et al. 2008).

The paper is structured as follows. The hierarchical modeling framework coupling a partial differential equation and a Bernoulli observation is described in Sect. 2. Bayesian inference for parameter estimation grounded on the AMIS algorithm and model selection are also presented in this methodological section. The results obtained from surveillance data for Xf in the case study (Corsica) are provided in Sect. 3. In Sect. 4, we summarize and discuss our work.

## The mechanistic-statistical approach

### Process model

Models based on parabolic partial differential equations have often been used to describe biological invasions (Skellam 1951; Shigesada et al. 1995; Shigesada and Kawasaki 1997; Okubo 1980). Here, we are interested in the invasion of a pathogen, that spreads in a domain $\varOmega$ included in ${\mathbb {R}}^2$. We assume that there is only one single introduction point in time and space that triggered the invasion and that eventual subsequent introductions have negligible effects on the dynamics and are therefore not incorporated into the model. Furthermore, to account for spatial heterogeneity in the reproduction regime of the pathogen, we divide $\varOmega$ into two sub-domains, say $\varOmega _{1}$ and $\varOmega _{2}$, such that $\varOmega =\varOmega _{1}\cup \varOmega _{2}$, $\varOmega _{1}\cap \varOmega _{2}=\emptyset$ and different growth terms apply to $\varOmega _{1}$ and $\varOmega _{2}$.

More formally, the spread of the pathogen is described by a coupled model governing the probability $u(t,{\mathbf {x}})$ of a host located at site ${\mathbf {x}}=(x_1,x_2)\in \varOmega$ to be infected at time *t*. This model is grounded on two particular types of parabolic partial differential equations: (i) a reaction–diffusion equation in $\varOmega _1$ where the growth is logistic (Verhulst 1838) and (ii) a diffusion–absorption equation in $\varOmega _2$ where only dispersal and death events occur. The probability $u(t,{\mathbf {x}})$ satisfies:1where $D>0$ is the diffusion coefficient; *b* corresponds to the intrinsic growth rate of the pathogen infection in $\varOmega _1$; $K\in (0,1]$ is a plateau for the probability of infection (i.e. an analogue to the carrying capacity of the environment); $\alpha$ is the decrease rate of the infection in $\varOmega _2$; $\varDelta =\dfrac{\partial ^2}{\partial x_1^2}+\dfrac{\partial ^2}{\partial x_2^2}$ is the 2-dimensional diffusion operator of Laplace;  is the characteristic function taking the value 1 if ${\mathbf {x}}\in \varOmega _{i}$ and 0 otherwise; $\tau _0\in {\mathbb {R}}$ is the introduction time of the pathogen. As explained in the introduction, the sub-domains $\varOmega _1$ and $\varOmega _2$ are defined by thresholding a spatial function, say *T*, with the threshold value ${{\tilde{T}}}$ that is hold fixed: $\varOmega _1=\varOmega _1(T,\tilde{T})=\{{{\mathbf {x}}}\in \varOmega :T({{\mathbf {x}}})> {{\tilde{T}}}\}$ and $\varOmega _2=\varOmega _2(T,{{\tilde{T}}})=\{{{\mathbf {x}}}\in \varOmega :T({\mathbf x})\le {{\tilde{T}}}\}$.

In our framework, the initial condition $u_0$ models the introduction of the pathogen in the study domain. Here, the introduction represents the initial phase of the outbreak corresponding to the arrival of the pathogen and its local establishment. Thus, $u_0$ is not expressed as a Dirac delta function but as a kernel function centered around the central point of the introduction ${\tilde{\mathbf {x}}}_0=({\tilde{x}}_0,\tilde{y}_0)\in \varOmega$. More precisely, the probability of a host at $\mathbf x$ to be infected at $\tau _0$ satisfies:

2 $$\begin{aligned} u_0(\mathbf{x})=p_0 \exp \left( -\dfrac{||{\mathbf {x}}-{\tilde{\mathbf {x}}}_0 ||^2}{2\sigma ^2}\right) \end{aligned}$$

where $$p_0$$ is the infection probability at $$(\tau _0,{\tilde{\mathbf {x}}}_0)$$, $$\sigma ^2=\frac{r_0^2}{q}$$, *q* is the 0.95-quantile of the $$\chi ^2$$ distribution with two degrees of freedom, and $$r_0$$ is the *radius* of the kernel. Thus, at $$\tau _0$$, if we neglect border effects, 95% of the infected plants are located within the ball with center $${\tilde{\mathbf {x}}}_0$$ and radius $$ r _0$$. Assuming in addition reflecting conditions on the boundary $$\partial \varOmega $$ of $$\varOmega $$, the system of equations (1) is well-posed (Evans 1998). In addition, by constraining $$p_0$$ in [0, *K*], the principle of parabolic comparison (Protter, MH and Weinberger, HF 1967) implies that the solution of (1) is also in the interval [0, *K*].

#### Remark

We adopted a parsimonious approach consisting of modeling the probability of a host to be infected (i.e., the local quantity of infected host units over the local total quantity of host units) instead of the dynamics of the pathogen in the host population (i.e., the local quantities of susceptible, exposed, infectious and removed host units). This choice allowed us, in particular, to ignore eventual spatial heterogeneity in host abundance and to reduce the number of unknown parameters.

### Data model

Let $t_i\in {{\mathbb {R}}}$ denote the sampling time of host $i\in \{1,\ldots ,I\}$, $I\in {{\mathbb {N}}}^*$, ${\mathbf {x}}_i\in \varOmega$ its location and $Y_i\in \{0,1\}$ its sanitary status observed at time $t_i$ (1 for infected, 0 for healthy). Conditionally on *u*, *T* and $\{(t_i,{\mathbf {x}}_i):1\le i \le I\}$, the sanitary statuses $Y_i$, $i\in \{1,\ldots ,I\}$, are assumed to be independent random variables following Bernoulli distributions with success probability $u(t_i,{\mathbf {x}}_i)$:

3 $$\begin{aligned} Y_i \mid u,T,\{(t_i,{\mathbf {x}}_i):1\le i \le I\} \underset{\text {indep.}}{\sim }\text {Bernoulli}(u(t_i,{\mathbf {x}}_i)), \end{aligned}$$

where *u* depends on parameters *D*, *b*, *K*, $$\alpha $$, $$\tau _0$$, $${\tilde{\mathbf {x}}}_0$$, $$ r _0$$, $$p_0$$ and $${\tilde{T}}$$.

#### Remark

This simple data model could be modified to account for factors classically encountered in epidemiology, e.g. false-positive and false-negative observations, and spatial and temporal dependencies not accounted for in the process model. In the real case study tackled in this article, each observed host was sampled only once. In a case where hosts could be sampled several times, a temporal dependence should be introduced in the observation process to account for, e.g., the within-host persistence of the pathogen.

### Parameter estimation with an adaptive importance sampling algorithm

Inference about the parameter vector $\varTheta =(D,b,K,\alpha ,\tau _0,{\tilde{\mathbf {x}}}_0, r _0,p_0)$ is made in the Bayesian framework, which technically consists in assessing the posterior distribution $[\varTheta |Y]$ of $\varTheta$ conditional on sanitary statuses $Y=\{Y_i:1\le i\le I\}$. The parameter ${\tilde{T}}$ will be treated later in Sect. 2.4 via model selection. Philosophically, a posterior probability is to be interpreted as a coherent judgment quantifying a subjective degree of uncertainty (Lindley 2006).

In what follows, we will keep using Gelfand’s bracket notations for probability distributions (Gelfand and Smith 1990). The posterior distribution of the unknown, hereafter dubbed $\varTheta$, is derived by Bayes’ rule:

$$\begin{aligned} {[}\varTheta |Y] = \frac{[Y|\varTheta ]\times [\varTheta ]}{[Y]}, \end{aligned}$$

where

$[Y|\varTheta ]$ is the conditional distribution of the data *Y* given the unknown $\varTheta$ (i.e. the likelihood function of the model) that satisfies [using Eq. (3)]:

4 $$\begin{aligned} {[}Y|\varTheta ] = \prod _{i=1}^I u(t_i,{\mathbf {x}}_i)^{Y_i}(1-u(t_i,{\mathbf {x}}_i))^{1-Y_i}; \end{aligned}$$

$[\varTheta ]$ is the prior distribution of $\varTheta$ that depends on the application and that will be specified in Sect. 3; the distribution of *Y*, $[Y]=\int [Y|\varTheta ][\varTheta ]d\varTheta$, may be a formidable integral, depending on the dimension of the unknown $\varTheta$. However, modern Bayesian algorithms (Brooks 2003) avoid its computation by making recourse to Monte Carlo techniques only based on the un-normalized probability function $[Y|\varTheta ]\times [\varTheta ]$. Yet, the computation of $[Y|\varTheta ]$ itself requires the value of the solution *u* of Eq. (1) for any valid parameter vector $\varTheta$. This equation admits a unique solution for any fixed and valid $\varTheta$, but cannot be solved analytically. Hence, we make recourse to a standard finite-element method with the software "Freefem++" (Hecht 2012); see Sect. 2.5.

For the mechanistic-statistical model defined above, the posterior distribution $[\varTheta |Y]$ cannot be expressed analytically due to its intractable normalizing constant, but one can draw a sample under this distribution using an adequate algorithm for Bayesian inference. The so-called posterior sample $[\varTheta |Y]$ is then used to numerically characterize all that we know about $\varTheta$ after data assimilation. Here, we use the adaptive multiple importance sampling (AMIS; Cornuet et al. 2012) algorithm, that consists of iteratively generating parameter vectors under an adaptive proposal distribution and assigning weights to the parameter vectors. To design efficient importance sampling algorithms, the auxiliary proposal distribution should be chosen as close as possible to the posterior distribution. However, the posterior distribution being unknown, the crucial choice of the proposal is a difficult task (Gelman et al. 1996; Roberts et al. 1997). The main aim of the AMIS algorithm is to overcome this difficulty by tuning the coefficients of the proposal distribution picked in a parametric family of distributions, generally the Gaussian one, at the end of each iteration.

In this framework, at each iteration, new coefficient values for the proposal distribution are determined using the current weighted posterior sample (Bugallo et al. 2015), then the posterior sample is augmented by generating new replicates from the newly tuned proposal distribution and the weights of the cumulated posterior sample are recomputed. The algorithm can be described as follows:

1. Set initial values $\mu _0$ and $\varSigma _0$ for the mean vector and the variance matrix of the multi-normal proposal distribution ${\mathcal {N}}(\mu _0,\varSigma _0)$, whose probability density function is denoted by $\varTheta \rightarrow g_{\mu _0,\varSigma _0}(\varTheta )$.
2. At iteration $$m=1,\cdots ,M$$,
  - Generate a new sample $\{\varTheta _m^l:l=1\cdots ,L\}$ from the proposal distribution ${\mathcal {N}}(\mu _{m-1},\varSigma _{m-1})$.
  - Compute the un-normalized importance weights for the new sample as in Eq. (5), and update the un-normalized weights for the previously generated samples as in Eq. (6):
    5 $$\begin{aligned} {\tilde{w}}_m^l&= \dfrac{[Y|\varTheta _m^l]\times [\varTheta _m^l]}{\dfrac{1}{m}\sum \limits _{j=1}^{m}g_{\mu _{j-1},\varSigma _{j-1}}(\varTheta _m^l)}, \,\, l=1,\cdots ,L \end{aligned}$$
    6 $$\begin{aligned} {\tilde{w}}_\varepsilon ^l&= \dfrac{[Y|\varTheta _\varepsilon ^l]\times [\varTheta _\varepsilon ^l]}{\dfrac{1}{m}\sum \limits _{j=1}^{m}g_{\mu _{j-1},\varSigma _{j-1}}(\varTheta _\varepsilon ^l)}, \,\, \varepsilon =1,\cdots ,m-1,\,\,l=1,\cdots ,L, \end{aligned}$$
    where $g_{\mu _{j-1},\varSigma _{j-1}}$ is the probability density function of the multi-normal distribution with mean vector $\mu _{j-1}$ and variance matrix $\varSigma _{j-1}$.
  - Normalize the weights:
    $$\begin{aligned} w_\varepsilon ^l=\dfrac{{\tilde{w}}_\varepsilon ^l}{\sum \limits _{i=1}^{m}\sum \limits _{j=1}^{L}{\tilde{w}}_i^j},\,\,\varepsilon =1,\cdots ,m,\,\,l=1,\cdots ,L. \end{aligned}$$
  - Adapt coefficient values for the next proposal distribution as follows:
    $$\begin{aligned} \mu _m&=\sum \limits _{l=1}^{L}\sum \limits _{\varepsilon =1}^{m}w_\varepsilon ^l \varTheta _\varepsilon ^l \\ \varSigma _m&=\sum \limits _{l=1}^{L}\sum \limits _{\varepsilon =1}^{m}w_\varepsilon ^l ( \varTheta _\varepsilon ^l-\mu _\varepsilon )(\varTheta _\varepsilon ^l-\mu _\varepsilon )^t. \end{aligned}$$

The AMIS algorithm provides a weighted posterior sample $\{\{\varTheta _m^l,w_m^l\}_{l=1}^L\}_{m=1}^M$ of size *ML*, which provides an empirical approximation of the posterior distribution $[\varTheta |Y]$. Conditions leading to the convergence in probability of the posterior mean of any function (integrable with respect to the posterior distribution) of the parameters are described in Cornuet et al. (2012) and are satisfied in our case.

If in practice, the convergence of AMIS to the true posterior cannot be numerically demonstrated (because the true posterior is not known), one can assess its stabilization

by evaluating the variation in the following deviation measure between the assessments of the posterior distribution at iteration $m-1$ and $m>1$:

$$\begin{aligned} {{\mathcal {M}}}_{{\mathcal {G}}}(m-1,m)=\underset{c\in {{\mathcal {G}}}}{\max } |p_m(c)-p_{m-1}(c)|, \end{aligned}$$

where $p_m(c)$ denotes the assessment at iteration *m* of the posterior probability that $\varTheta$ is in the sub-domain $c\subset {{\mathbb {R}}}^8$ of the parameter space, i.e.and ${\mathcal {G}}$ is a partition of a sub-space of the parameter space. The definition of ${\mathcal {G}}$ depends on the application and will be given in the Results section.

We implemented AMIS in the "R" statistical software, except for solving the PDE, which was performed by calling the "Freefem++" software from "R" each time a new parameter vector was proposed. Parallel computation was performed: the estimation procedure for a fixed value of ${\tilde{T}}$ took approximately 1.75  days with $(M,L)=(50,10^4)$ and the use of 100 computer cores.

### Choice of ${{\tilde{T}}}$ with a model selection procedure

Implementation constraints concerning the partition of the study domain which depends on the threshold ${{\tilde{T}}}$, led us to proceed by two separate steps: (i) to infer model parameters for different fixed values of ${{\tilde{T}}}$ and, then, (ii) to select the value of ${{\tilde{T}}}$ having the largest support of data (this amounts to selecting a model within a class of models characterized by $\tilde{T}$). Thus, for each element ${{\tilde{T}}}_a$ in $\{\tilde{T}_1,\ldots ,{{\tilde{T}}}_A\}\subset {\mathbb {R}}^A$, $A\in {\mathbb {N}}^*$, we carried out the estimation procedure described in Sect. 2.3 by instancing ${{\tilde{T}}}$ at the value $\tilde{T}_a$ and letting it fixed. Then, the best value of ${{\tilde{T}}}$ is chosen by minimizing some criteria classically used for model selection: here we rely on the Bayesian Information criterion (BIC; Schwarz et al. 1978), two Deviance information criteria (DIC; Spiegelhalter et al. 2002; Gelman et al. 2003) and a predictive Information Criterion (IC; Ando 2011). We use different selection criteria in order to report the variability of the selected ${{\tilde{T}}}$ when different hypotheses are made about which the best model is, if any.

The BIC satisfies:

7 $$\begin{aligned} \text {BIC} =-2\log [Y|{\hat{\varTheta }}]+k \log I, \end{aligned}$$

where *I* is the sample size, *k* is the number of model parameters (in our setting, *k* is the same for all the models), and ${\hat{\varTheta }}$ is the maximum likelihood estimate of the parameter vector $\varTheta$ in the support ${\mathcal {S}}(\varTheta ;{{\tilde{T}}}_a)$ of $\varTheta$ defined by the prior distribution (in our setting, this support depends on the fixed value ${{\tilde{T}}}_a$ of ${{\tilde{T}}}$):

$$\begin{aligned} {\hat{\varTheta }}=\underset{\varTheta \in {\mathcal {S}}(\varTheta ;{{\tilde{T}}}_a)}{\text {argmax}}[Y|\varTheta ]. \end{aligned}$$

The DIC satisfies:

8 $$\begin{aligned} \text {DIC} ={\mathcal {{\bar{D}}}}+p_\text {eff}, \end{aligned}$$

where ${{\mathcal {\bar{D}}}}$ is the posterior mean of the deviance ${\mathcal {D}}(\varTheta )=-2\log [Y|\varTheta ]+C$ (where *C* is a constant that cancels out when one compares different models) and $p_\text {eff}$ is the effective number of parameters of the model. The difference in the two versions of the DIC considered here lies in the calculation of $p_\text {eff}$. In the first version proposed by Spiegelhalter et al. (2002),

9 $$\begin{aligned} p_\text {eff}=p_{\mathcal {D}}=\mathcal {\bar{D}}-{\mathcal {D}}({\bar{\varTheta }}), \end{aligned}$$

where ${\bar{\varTheta }}$ is the posterior mean of $\varTheta$: . In the second version proposed by Gelman et al. (2003),10where  is the posterior variance of ${\mathcal {D}}(\varTheta )$. The IC of Ando (2011), which is supposed to solve over-fitting issues, satisfies:

11 $$\begin{aligned} \text {IC} ={{\mathcal {\bar{D}}}}+2p_{\mathcal {D}}:=3{{\mathcal {\bar{D}}}} -2{\mathcal {D}}({\bar{\varTheta }}). \end{aligned}$$

In practice, the different terms appearing in the four criteria, namely ${{\hat{\varTheta }}}$, ${\bar{\varTheta }}$, ${{\mathcal {\bar{D}}}}$ and , are replaced by their empirical values using the weighted posterior sample $\{\{\varTheta _m^l,w_m^l\}_{l=1}^L\}_{m=1}^M$ provided by the application of the AMIS algorithm.

### Numerical equation solving

For the application, computations for solving the PDE were carried out with the software "Freefem++"  (Hecht 2012). A Finite Element Method was used. The non-linearity has been treated with a Newton-Raphson algorithm applied to the variational formulation of Equation (1), by instancing the criterion of convergence at the value $10^{-10}$. The solution was approximated by a piecewise linear and continuous function. The time resolution was based on an adaptive step size using a backward Euler method. Supplementary Figure S1 shows the spatial discretization composed of 4791 nodes that has been used in the application in Sect. 3. With this mesh, the average computation time for one simulation is 55 s. We explored the effect of the spatial discretization by comparing the numerical solutions of the equation obtained with the 4791 nodes mesh and with a finer mesh composed of 10703 nodes. The solutions were computed for the set of parameters corresponding to the posterior maximum (Supplementary Material S4 shows the time continuous dynamics for this set of parameters). Supplementary Figure S2 shows very close simulation results for both meshes. Moreover, we investigated the numerical error of system 1 by using the indicator, norm $||u||_{H^2}$ which is classically considered to control the $H^1$-error (Allaire 2008). Using the mesh composed of 4791 nodes leads to a numerical error around 0.02 corresponding to a satisfying accuracy for our application.

## Application to the dynamics of *Xylella fastidiosa* in South Corsica

### Surveillance data

**Fig. 1:**
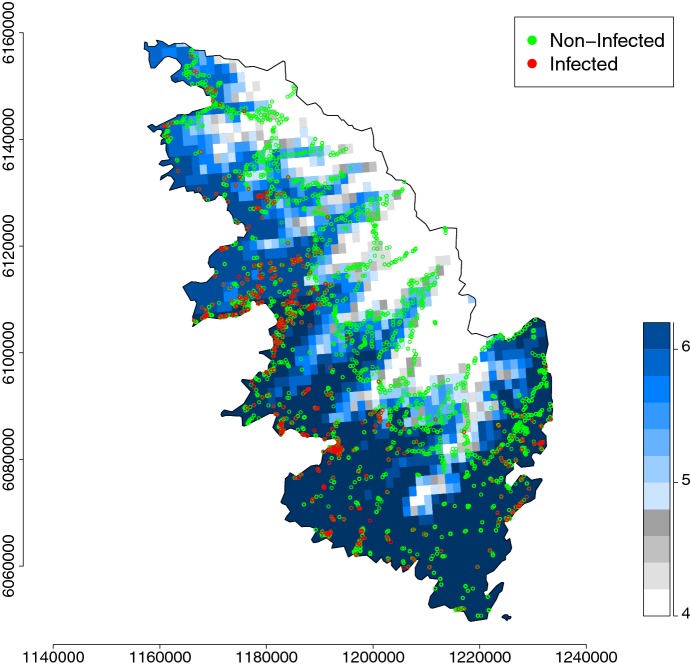
Locations of plants, sampled from July 2015 to May 2017, that have been detected as positive (red dots) or negative (green dots) to *Xylella fastidiosa* in South Corsica, France, and map of the average of the daily minimum temperature (in Celsius degrees) over January and February reconstructed over a grid with spatial resolution of 1$\times$1km (blue–white color palette) (color figure online)

For this application, we use spatio-temporal binary data on the presence of *Xylella fastidiosa* (Xf) collected in South Corsica, France, from July 2015 to May 2017. Over this period, approximately 8000 plants were sampled, among which 800 have been diagnosed as infected (with a real-time polymerase chain reaction (PCR) technique; Denancé et al. 2017b). Available data for each sampled plant are its spatial coordinates, its sampling date (which is unique) and its health status at the sampling date. Coordinates and health statuses at the sampling times are shown in Fig. 1.

### Model specifications

**Fig. 2:**
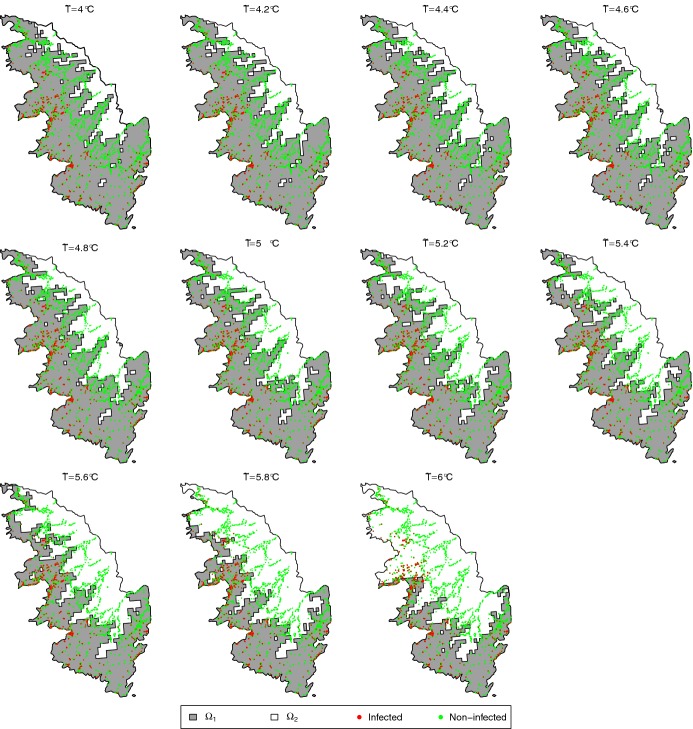
Partition of the study domain $\varOmega$ into the sub-domains $\varOmega _1$ and $\varOmega _2$ with respect to the value of ${{\tilde{T}}}$ in $\{4.0,4.2,4.4,\ldots ,6.0\}$, in Celsius degrees. Red and green dots give the locations of infected and non-infected samples (color figure online)

As mentioned in the introduction, we use temperature data to divide the spatial domain into two sub-domains. We exploit a freely available database (PVGIS $\copyright$ European Communities, 2001–2008) providing, in particular, monthly averages of the daily minimum temperature reconstructed over a grid with spatial resolution of 1$\times$1km (Huld et al. 2006); these monthly averages correspond to the period 1995-2003, but we used them as references over the period covered by our models. We use these data to build the average of the daily minimum temperature over January and February, say $T({\mathbf {x}})$ for any location ${\mathbf {x}}$; see Fig. 1. $T({\mathbf {x}})$ is then used to split the study domain into two parts: one part where $T({\mathbf {x}})\le {\tilde{T}}$ and the growth of Xf is hampered by cold winter temperatures, and the other part where $T({\mathbf {x}})>{\tilde{T}}$ and the growth of Xf is not hampered. The threshold value ${\tilde{T}}$ will be selected in the set $\{4.0,4.2,4.4,\ldots ,6.0\}$, in Celsius degrees. Panels of Fig. 2 display the partitioning of the study domain induced by the different values of ${\tilde{T}}$.

The prior distribution for $\varTheta$ combines vague uniform distributions and Dirac distributions:where $|\varOmega _1|$ is the area of $\varOmega _1$ and $\text {Dirac}_b(B)$ is equal to 1 if $B=b$, and 0 otherwise. The Dirac distribution for ${{\tilde{T}}}$ was chosen to deal with implementation issues explained in Section 2.4. We chose Dirac prior distributions for $r_0$ and $p_0$ in the aim of precisely defining what is an *introduction* (see Section 2.1) and imposing the same intensity level and spatial extent for the introduction in all the models in competition. For *D*, *b*, *K* and $\alpha$, we specified vague uniform priors satisfying constraints of positivity. In addition, the plateau *K* had to be less than 1, as indicated in Sect. 2.1. For the introduction time $\tau _0$, we chose a uniform distribution between $-1000$ months and 0 month before the first detection of Xf in South Corsica. Note that, using a temporal model and aggregated data, Soubeyrand et al. (2018) inferred an introduction date around $-360$ months before the first detection of Xf in South Corsica. Finally, the introduction location ${\tilde{\mathbf {x}}}_0$ was supposed to be uniformly distributed in $\varOmega _1$, the sub-domain where the conditions are favorable for the expansion of Xf.

### Selection of the temperature threshold

**Fig. 3:**
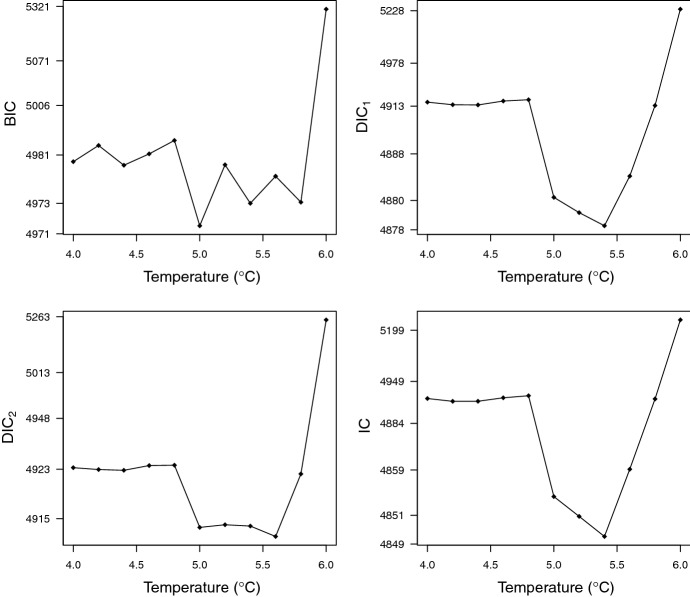
Values of the four selection criteria (BIC, $\text {DIC}_1$ of Spiegelhalter et al. (2002), $\text {DIC}_2$ of (Gelman et al. 2003), IC of Ando (2011)) for different thresholds of temperature ${\tilde{T}}$ ranging from 4 to 6 $^\circ \hbox {C}$. Non-linear transformations of the y-axis were applied to facilitate the identification of the lowest values of the criteria

The spatio-temporal models corresponding to different values of ${\tilde{T}}$ ranging from 4 to 6$^\circ \hbox {C}$ were fitted to data using the estimation approach presented in Sect. 2.3 (with $(M,L)=(50,10^4)$) and were compared with the four selection criteria introduced in Sect. 2.4. The values of the criteria are displayed in Fig. 3. The smallest BIC value was obtained for ${\tilde{T}}=5.0 \,^\circ \hbox {C}$. The smallest DIC value based on the computation proposed by Spiegelhalter et al. (2002) and the smallest IC values were obtained for ${\tilde{T}}=5.4\,^\circ \hbox {C}$. The smallest DIC value based on the computation proposed by Gelman et al. (2003) was obtained for ${\tilde{T}}=5.6\,^\circ \hbox {C}$. Except the BIC, which only measures the adequacy between the model and data at the posterior mode of the parameter vector, each of the three other criteria takes quite close values around ${\tilde{T}}=5.4\,^\circ \hbox {C}$ (typically from 5.0 to 5.6  $^\circ \hbox {C}$). In what follows, we present the results obtained with the model corresponding to the threshold ${\tilde{T}}=5.4\,^\circ \hbox {C}$, which is a satisfying compromise.

### Stabilization of the AMIS algorithm

**Fig. 4:**
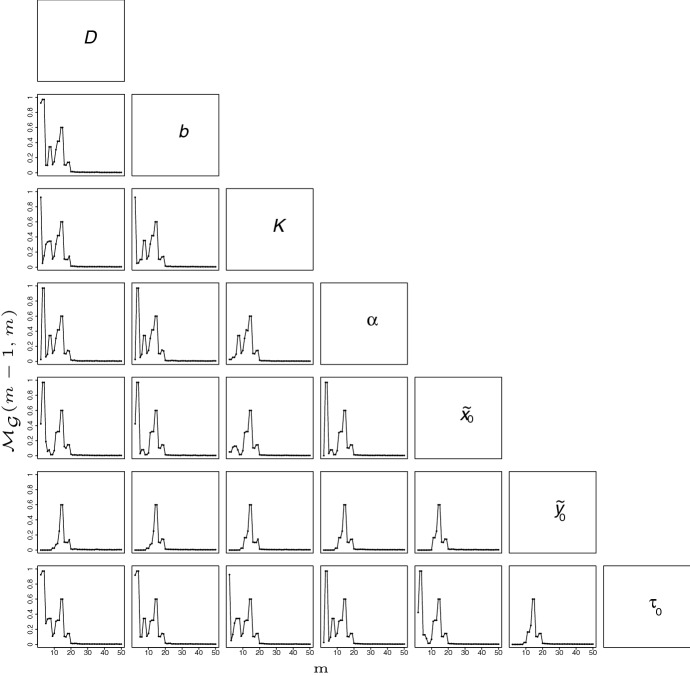
Variation in the deviation measure ${\mathcal M}_{{\mathcal {G}}}(m-1,m)$ between the assessments of the posterior distribution at iteration $m-1$ and $m>1$ of the AMIS algorithm. ${{\mathcal {M}}}_{{\mathcal {G}}}(m-1,m)$ is plotted for different partitions ${\mathcal {G}}$ allowing the assessment of the stabilization of all the 2D posterior distributions of parameters *D*, *b*, *K*, $\alpha$, ${{\tilde{x}}}_0$, ${{\tilde{y}}}_0$ and $\tau _0$

**Fig. 5:**
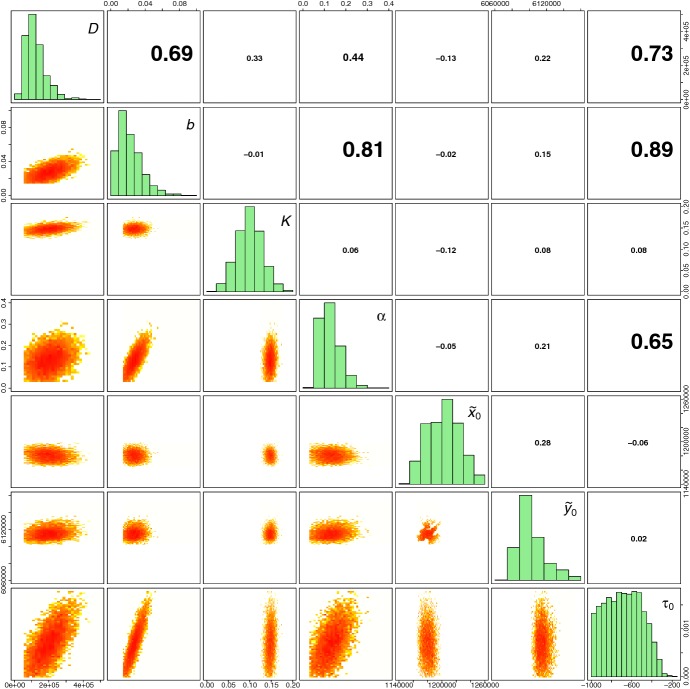
Marginal posterior distributions of parameters (panels in the diagonal) and 2D posterior distributions of parameters over the 60$\times$60 grids described in Sect. 3.4 (panels in the lower triangle). Figures in the upper triangle panels provide correlation coefficients (the larger the text size, the stronger the correlation)

Figure 4 gives the variation in ${{\mathcal {M}}}_{\mathcal G}(m-1,m)$ for different partitions ${\mathcal {G}}$ allowing us to assess the stabilization of all the 2D posterior distributions of parameters (see Sect. 2.3 for the definition of the deviation measure ${{\mathcal {M}}}_{{\mathcal {G}}}$). For each pair of parameters, ${\mathcal {G}}$ was defined as the set of infinite cylinders with rectangular bases whose orthogonal projection in the 2 dimensions of interest forms a 60$\times$60 regular rectangular grid. In each dimension of interest, the endpoints of the grid were set at the minimum and maximum values of the corresponding parameter having a weight $w_{M}^l$ larger than $10^{-5}$ (the 2D posterior distributions over these 60$\times$60 grids are displayed in Fig. 5). Figure 4 shows the stabilization of all the 2D posterior distributions after iteration 21.

### Posterior distribution of parameters

**Fig. 6:**
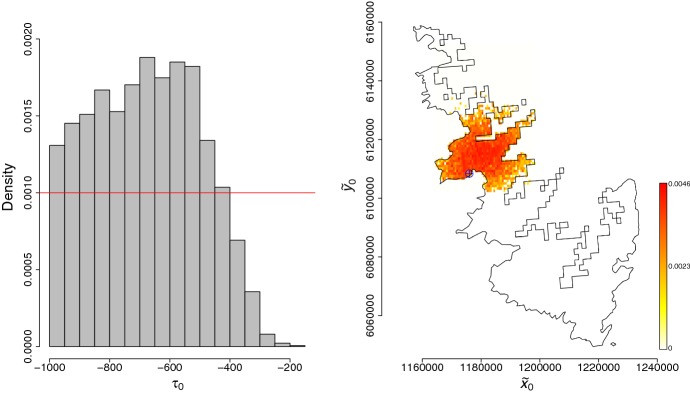
Posterior distributions of the introduction time $\tau _0$ (histogram) and the introduction point $\tilde{\mathbf{x}}_0$ (color palette). The prior for $\tau _0$ was uniform over $[-1000,0]$ (red line). The value of $\tilde{\mathbf{x}}_0$ having the largest weight in AMIS is indicated by a blue cross. The prior for $\tilde{\mathbf{x}}_0$ was uniform over the space delimited by the contours (color figure online)

**Fig. 7:**
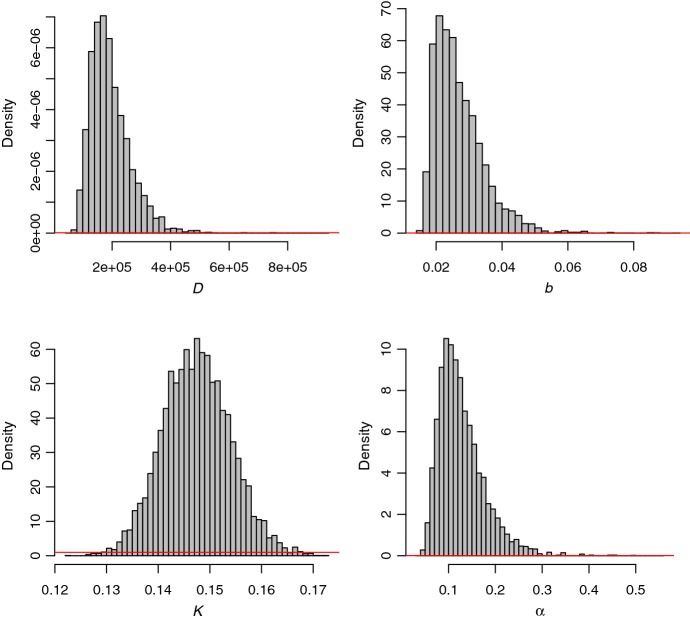
Marginal posterior distributions of *D*, *b*, *K* and $\alpha$ (histograms) and corresponding prior distributions (red lines) over the supports covered by the posteriors (color figure online)

Marginal and 2D posterior distributions of parameters are displayed in Figs. 5, 6 and 7. The introduction of Xf tends to be relatively ancient (posterior median: $-680$ months before July 2015, i.e. introduction around 1959; posterior mean $-681$ months) but also relatively uncertain (posterior standard deviation: 179 months). This uncertainty has to be regarded in the light of the relatively high posterior correlation between $\tau _0$ and the reaction–diffusion–absorption parameters *D*, *b* and $\alpha$. Acquiring knowledge about *D*, *b* and $\alpha$ could help in eliciting informative priors for these parameters and obtain a less uncertain estimation of the introduction date. Based on our analysis, the introduction probably occurred around Ajaccio or the surrounding municipalities in the East, North and North-East (Right panel of Fig. 6). Figure 7 and Table 1 show posterior distributions and statistics of *D*, *b*, *K* and $\alpha$. In particular, we observe that the plateau for the probability of infection is around 15%. This relatively low estimate has to be considered with caution. First, it is relative to the population of plants that have been sampled. Second, it ignores the risk of false-negative observations. The inference about the diffusion parameter *D* allowed us to assess the length of a straight line move of the pathogen during a time unit, namely the month. This length is given by Eq. (12) (Turchin 1998; Roques et al. 2016):

12 $$\begin{aligned} D =\dfrac{(\text {length of a straight line move during one time step})^2}{4\times \text {duration of the time step}}, \end{aligned}$$

and has a posterior median equal to 155 meters per month (posterior mean: 155; posterior standard deviation: 27). These figures correspond to the move of the pathogen with different means, in particular via insects and transportation of infected plants, which are both modeled by the diffusion operator in Eq. (1).

### Goodness-of-fit of the model

To check the adequacy between the selected model and observed data, we measured the accuracy of the probabilistic predictions provided by the model by using the Brier score (BS) (Brier 1950). This score is the mean of the square differences between (i) the observed health statuses $Y_i^{\text {obs}}$, $i=1,\ldots ,I$(which is a realization of $Y_i$ and takes values in $\{0,1\}$), and (ii) the corresponding probabilities of infection $u(t_i,{\mathbf {x}}_i)$, which depend on $\varTheta$:

13 $$\begin{aligned} \text {BS}=\dfrac{1}{I}\sum _{i=1}^I\big ({Y_i^{\text {obs}}}-u(t_i,{\mathbf {x}}_i)\big )^2. \end{aligned}$$

The Brier score varies between 0 and 1; lower the Brier score, better the goodness-of-fit; a systematic prediction of 0.5 leads to a Brier score equal to 0.25, which can be viewed as a threshold above which the model is clearly inadequate. In our application, the posterior median of BS is 0.0829 (95%-posterior interval: [0.0827,0.0830]).

**Table 1:** Posterior medians, means and standard deviations of parameters of the reaction–diffusion–absorption equation

Parameter	Unit	Median	Mean	Standard deviation
*D*	$\hbox {m}^2$ $\hbox {month}^{-1}$	$1.8\times 10^{5}$	$2.0\times 10^{5}$	$0.7\times 10^{5}$
*b*	$\hbox {month}^{-1}$	0.026	0.027	0.008
*K*	probability	0.147	0.148	0.007
$\alpha$	$\hbox {month}^{-1}$	0.12	0.13	0.05

The probabilistic predictions provided by the model can also be compared to simple but data-informed predictions via the Brier skill score (BSS):

$$\begin{aligned} \text {BSS} = 1-\frac{\text {BS}}{\text {BS}_\text {ref}}, \end{aligned}$$

where $\text {BS}_\text {ref}$ is the Brier score for a reference forecast. The BSS takes values between $-\infty$ and 1; A positive (resp. negative) BSS value indicates that the model-based prediction is more (resp. less) accurate than the reference forecast. The most common reference forecast is the so-called *climatology* forecast (Mason 2004) that is the mean ${\bar{Y}}^{\text {obs}}$ of $\{{Y_i^{\text {obs}}}:i=1,\ldots ,I\}$: $\text {BS}_\text {ref}=(1/I)\sum _{i=1}^I({Y_i^{\text {obs}}-\bar{Y}^{\text {obs}}})^2$. In our application, the posterior median of BSS is 0.031 and its 95%-posterior interval is [0.029, 0.032], which is entirely above zero. Hence, the model-based prediction tends to be significantly more accurate than the *climatology* forecast.

**Fig. 8:**
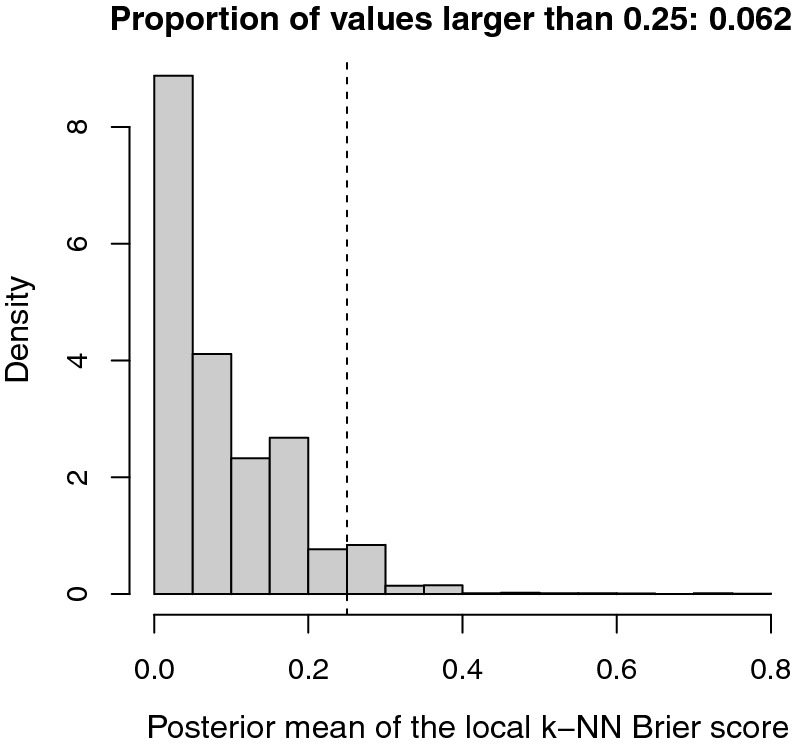
Distribution of the posterior means of the local Brier scores with $k=20$. The dashed line gives the 0.25 threshold

**Fig. 9:**
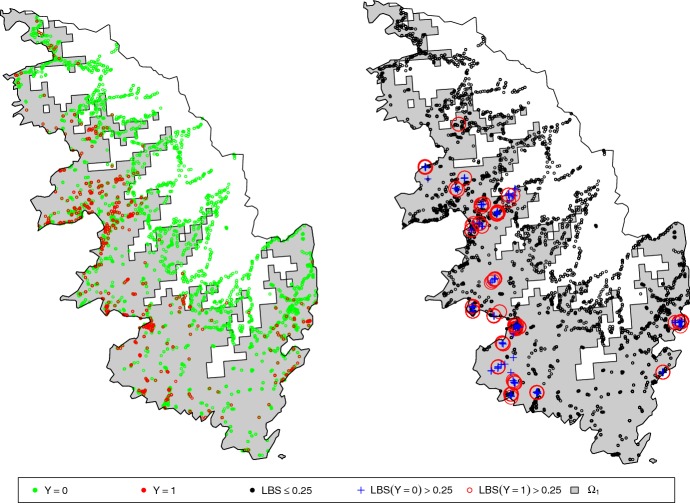
Locations of samples diagnosed as positive and negative to *Xylella fastidiosa* (left) and samples with different levels of the local Brier score with $k=20$ (right; black circles: $\hbox {LBS}_{{20}}(i)\le 0.25$; blue crosses: $Y_i^{\text {obs}}=0$ and $\hbox {LBS}_{{20}}(i)> 0.25$; red circles: $Y_i^{\text {obs}}=1$ and $\hbox {LBS}_{{20}}(i)> 0.25$). The gray surface gives the extent of $\varOmega _1$ (color figure online)

We extended the goodness-of-fit analysis by building and analyzing a local Brier score that allows us to check the adequacy of the model across space. The local Brier score (LBS) computed at the location of observation $i\in \{1,\ldots ,I\}$ is defined as a mean over the *k*-nearest neighbors:

14 $$\begin{aligned} \text {LBS}_k(i)=\frac{1}{k+1}\sum _{i'\in \{i\}\cup {\mathcal {V}}_k(i)}\big ({Y_{i'}^{\text {obs}}}-u(t_{i'},{\mathbf {x}}_{i'})\big )^2, \end{aligned}$$

where ${\mathcal {V}}_k(i)$ is the set of indices in $\{1,\ldots ,I\}$ corresponding to the $k>0$ observations nearest to ${\mathbf {x}}_i$ with respect to the Euclidean distance in . Figure 8 gives the distribution of the posterior means of the local Brier scores (Remark: each $\text {LBS}_k(i)$ has a posterior mean because it depends on $\theta$ via the function *u*). 6.2% of these scores are above 0.25, which is a rather small percentage. Figure 9 displays locations where the LBS is larger than 0.25 with $k=20$ (Supplementary Figure S3 provides similar information for *k* equal to 50, 100 and 150). This figure also indicates whether observations with LBS>0.25 were detected as positive or negative to Xf. None of the observations with LBS>0.25 are in $\varOmega _2$ where the growth of the pathogen is negative. Thus, discrepancies between data and the model are limited to $\varOmega _1$. In addition, in general, model discrepancies for positive samples and negative samples are located approximately at the same places. Therefore, there might be some spatially abrupt changes in the rate of infection that are not represented by our aggregated model.

## Discussion

Since the detection of Xf in Europe, several modeling approaches have been implemented to provide more insights on the spread of this invasive pathogen in European environments (Strona et al. 2017; White et al. 2017; Bosso et al. 2016; Godefroid et al. 2018; Soubeyrand et al. 2018; Martinetti and Soubeyrand 2018). In this paper, we mainly focus on dating and localizing the introduction of this invasive species. Nevertheless, inferring the parameters of the coupled reaction–diffusion–absorption equation is required since only post-introduction data are available. The conducted analyses using a Bayesian inference approach, tend to show that the introduction of Xf in South Corsica occurred probably near Ajaccio around 1959 (95%-posterior interval: [1933, 1986]), long time before its first detection. Our estimation of the introduction time is relatively consistent with the results obtained by Denancé et al. (2017a) who assessed the introduction of the two main strains found in Corsica around 1965 and 1980, respectively, using a phylogenetic approach. Likewise, our estimation is compatible to the result of Soubeyrand et al. (2018), who dated the introduction around 1985 (95%-posterior interval: [1978, 1993]) with a statistical analysis of temporal data (indeed, the posterior intervals obtained from both analyses overlap). To obtain a more accurate estimation of the introduction date, at least two tracks could be followed: coupling the analysis of spatio-temporal surveillance data and genetic data, as discussed in Soubeyrand et al. (2018), and, as suggested in the result section, gaining knowledge about parameters *D*, *b* and $\alpha$ whose estimations are correlated with the estimation of the introduction date (such a knowledge could be incorporated into the prior distribution and could lead to a narrower posterior distribution of $\tau _0$).

To infer the posterior distribution of the parameter vector we proceed in two steps: (i) infer the parameters of the dynamics given the temperature threshold ${\tilde{T}}$ used for partitioning the study domain, and (ii) choose ${\tilde{T}}$ using different selection criteria. A possible extension of our work is to refine the definition of the spatial partition by not only using the minimum daily winter temperature but also other relevant environmental variables (Godefroid et al. 2018; Martinetti and Soubeyrand 2018). Thus, a parametric logistic regression function depending on these variables could be built for partitioning the study domain and its parameters should be jointly estimated with the other parameters. However, this perspective requires a faster estimation approach. Indeed, an important milestone towards an accurate inference about the parameter vector, is to accurately solve the partial differential equation, which requires non-negligible computation time. Fortunately, the AMIS algorithm is easily parallelized. However, jointly estimating the partition of the study domain (and not only selecting it as we did), would result on much larger computation times, especially if the partition depends on multiple spatial variables. To reduce the computational cost, approximating the input/output relation in the mechanistic model using meta-models necessitating less computer intensive calculations could be a valuable option, that could be incorporated in AMIS (Osio and Amon 1996; Giunta and Watson 1998). In particular, kriging meta-models show up to be an adequate solution for approximating deterministic models since they interpolate the observed or known data points (Simpson et al. 2001). An additional advantage that derives from the use of AMIS is that its tuning parameters are adapted across the algorithm iterations, contrary to the basic MCMC and the maximum likelihood (ML) approach frequently used in the mechanistic-statistical framework. It has however to be noted that AMIS has to be appropriately initialized, which can be relatively easily done in practice by evaluating the marginal posterior distributions over 1D grids. Still to regard with the computational cost, ML estimation could be an interesting option, even if the control of estimation uncertainty is more convincing in the Bayesian framework for a model such ours. Supplementary Section S3 and Figure S4 precisely investigate ML applied to our case study: using the Broyden-Fletcher-Goldfarb-Shanno (BFGS) algorithm for the maximization, the computation effort is reduced, but results tend to indicate that the optimization is stuck in local maxima. More complex optimization algorithms, such as the simulated annealing algorithm, could be applied to converge to a global maximum but much more computations would hence be required.

Obviously, the deterministic model [Eqs. (1–2)] that we proposed to describe the dynamics of the pathogen does not take into account all the epidemiological and environmental drivers of the dynamics. These drivers could be implicitly handled by replacing our model by a stochastic version that would result in more flexible realizations. Gonze et al. (2002) compared deterministic and stochastic models for circadian oscillations and showed that, in presence of noise in a small population, stochastic simulations are needed to get more realistic realizations. Although the population size for the case study of Xf is expected to be relatively large, stochastic population-dynamic models, from individual-based models (Renshaw 1993; Kareiva and Shigesada 1983) to aggregated models (Soubeyrand et al. 2009b), could allow to relax hypotheses made on the dynamics. In contrast, our parsimonious model, which only incorporates the main epidemiological and environmental drivers, provides a concise description of the dynamics of the pathogen, and can be fitted to data in a reasonable time span. The advantage of this approach is that it can be rapidly applied for endorsing a fast reaction after the detection of a new invasive pathogen.

Instead of replacing our model by a stochastic version, we could refine it by taking into account relevant supplementary epidemiological and environmental processes. For instance, the diffusion, the growth/decrease of the pathogen infection and the plateau for the infection probability (represented in our model by parameters *D*, *b*, $\alpha$ and *K*) could depend on the spatio-temporal distribution of insects transmitting the pathogen, host density, seasonality and other environmental factors. Incorporating such dependencies into the model and using sufficiently high-resolution maps for spatial factors could allow the modeling of rapid changes in the infection probability that have been observed in Sect. 3.6. This sort of model refinement probably requires, however, more data than we have for Xf. For example, mapping host-density for Xf is not an easy issue because of the large spectrum of host species and the large variability in species susceptibility. Similarly, estimating seasonal effects on the growth/decrease rate of the infection probability certainly requires a larger observation temporal window allowing the detection of seasonal trends (in our case study, observations, which are available during only 2 years long after the introduction, mostly give information on the accumulation of the disease across time, but not on within-year variations of the infection probability). Neglecting all these factors implies that our framework provides estimates of *efficient* parameters (e.g., we estimate an *efficient* diffusion coefficient because diffusion is *averaged* over time in our model, neglecting seasonality in the presence of insect vectors and in the transportation of plants).

An additional perspective for the framework that we proposed is the use of alternative representations of disease propagation. The homogeneous diffusion could be replaced by an heterogeneous diffusion as proposed above, but could also be replaced/augmented by a kernel-based term within an integro-differential equation (Bonnefon et al. 2014), a spatial contact model (Mollison 1977), a mixed dispersal kernel model (Clark et al. 1998), a stratified dispersal model (Shigesada et al. 1995) or a piecewise deterministic Markov process (Abboud et al. 2018). These approaches, allowing a finer quantification of local and long distance dispersal, are generally expected to yield better predictions (Higgins and Richardson 1999; Nathan et al. 2008; Fayard et al. 2009; Gilioli et al. 2013; White et al. 2017). For instance, White et al. (2017) model the spread of Xf in the (supposed) early stages of the invasion in Apulia, Italy, with a stratified dispersal approach. They predict that the long-distance dispersal component is a paramount driver of the rapid spread of the pathogen and has to be taken into account in the design of management strategies. They however advocate that field estimates of key parameters, such as infection growth rate, local and non-local dispersal parameters, should be estimated to decrease prediction uncertainty. The relatively simple framework that we propose precisely provides, using field data, estimates of such parameters and other quantities such as the temperature threshold, the date and the location of the pathogen introduction. Regarding the pathogen introduction, we assumed that there is only one introduction that triggered the invasion and that eventual subsequent introductions had negligible effects on the dynamics. In the aim of relaxing this assumption, stratified dispersal models and piecewise deterministic Markov processes (PDMP) discussed above can be designed to incorporate into the model not only long-distance dispersal but also multiple introductions. Distinguishing these two types of events from surveillance data is not easy in general, except if one has at disposal genetic data or contact tracing data, but can anyway be modeled separately with a mixture of two kernels (identifiability issues of the mixture components may however arise). Abboud et al. (2018) precisely discuss a PDMP embedding multiple introductions without implementing it in practice. This is one of the most attractive perspectives for furthering our work.

## Electronic supplementary material

Below is the link to the electronic supplementary material.

Supplementary material 1 (pdf 5321 KB)

Supplementary material 2 (mp4 2921 KB)

## References

[CR1] Abboud C, Senoussi R, Soubeyrand S, Azaïs R, Bouguet F (2018). Piecewise-deterministic Markov processes for spatio-temporal population dynamics. Statistical inference for piecewise-deterministic Markov processes.

[CR2] Allaire G (2008). Analyse numérique et optimisation.

[CR3] Anas O, Harrison UJ, Brannen PM, Sutton TB (2008). The effect of warming winter temperature on the severity of pierce’s disease in the appalachian mountains and piedmont of the southeastern United States. Plant Health Prog.

[CR4] Anderson RM, Donnelly CA, Ferguson NM, Woolhouse MEJ, Watt CJ, Udy HJ, Mawhinney S, Dunstan SP, Southwood TRE, Wilesmith JW, Ryan JBM, Hoinville LJ, Hillerton JE, Austin AR, Wells GAH (1996). Transmission dynamics and epidemiology of BSE in British cattle. Nature.

[CR5] Ando T (2011). Predictive Bayesian model selection. Am J Math Manag Sci.

[CR6] Andow D, Kareiva PM, Levin SA, Okubo A (1990). Spread of invading organisms. Landsc Ecol.

[CR7] Andow DA, Kareiva PM, Levin SA, Okubo A, Kim KC, McPheron BA (1993). Spread of invading organisms: patterns of spread. Evolution of insect pests: the pattern of variations.

[CR8] Baker HG (1991). The continuing evolution of weeds. Econ Bot.

[CR9] Berliner LM (2003). Physical-statistical modeling in geophysics. J Geophys Res Atmos.

[CR10] Bonnefon O, Coville J, Garnier J, Roques L (2014). Inside dynamics of solutions of integro-differential equations. Discrete Contin Dyn Syst B.

[CR11] Bosso L, Russo D, Febbraro MD, Cristinzio G, Zoina A (2016). Potential distribution of *Xylella fastidiosa* in Italy: a maximum entropy model. Phytopathol Mediterr.

[CR12] Boys RJ, Wilkinson DJ, Kirkwood TBL (2008). Bayesian inference for a discretely observed stochastic kinetic model. Stat Comput.

[CR13] Brier GW (1950). Verification of forecasts expressed in terms of probability. OPTmonthey Weather Rev.

[CR14] Brooks S (2003). Bayesian computation: a statistical revolution. Trans R Stat Soc Ser A.

[CR15] Bugallo MF, Martino L, Corander J (2015). Adaptive importance sampling in signal processing. Digit Signal Process.

[CR16] Chapman DS, White SM, Hooftman DA, Bullock JM (2015). Inventory and review of quantitative models for spread of plant pests for use in pest risk assessment for the EU Territory.

[CR17] Clark JS, Fastie C, Hurtt G, Jackson ST, Johnson C, King GA, Lewis M, Lynch J, Pacala S, Prentice C, Schupp EW, Webb T, Wyckoff P (1998). Reid’s paradox of rapid plant migration: dispersal theory and interpretation of paleoecological records. BioScience.

[CR18] Cornuet J, Marin JM, Mira A, Robert CP (2012). Adaptive multiple importance sampling. Scand J Stat.

[CR19] Costello M, Steinmaus S, Boisseranc C (2017). Environmental variables influencing the incidence of Pierce’s disease. Aust J Grape Wine Res.

[CR20] Denancé N, Cesbron S, Briand M, Rieux A, Jacques MA (2017a) Is *Xylella fastidiosa* really emerging in France? In: Costa J, Koebnik R (eds) 1st Annual conference of the EuroXanth—COST action integrating science on *Xanthomonadaceae* for integrated plant disease management in Europe, EuroXanth, Coimbra, Portugal, vol 7

[CR21] Denancé N, Legendre B, Briand M, Olivier V, Boisseson C, Poliakoff F, Jacques MA (2017). Several subspecies and sequence types are associated with the emergence of *Xylella fastidiosa* in natural settings in France. Plant Pathol.

[CR22] Evans LC (1998). Partial differential equations, graduate studies in mathematics.

[CR23] Fayard J, Klein EK, Lefèvre F (2009). Long distance dispersal and the fate of a gene from the colonization front. J Evol Biol.

[CR24] Feil H, Purcell AH (2001). Temperature-dependent growth and survival of *Xylella fastidiosa* in vitro and in potted grapevines. Plant Dis.

[CR25] Feil H, Feil WS, Purcell AH (2003). Effects of date of inoculation on the within-plant movement of *Xylella fastidiosa* and persistence of Pierce’s disease within field grapevines. Phytopathology.

[CR26] Fisher RA (1937). The wave of advance of advantageous genes. Ann Eugen.

[CR27] Gatenby RA, Gawlinski ET (1996). A reaction–diffusion model of cancer invasion. Cancer Res.

[CR28] Gelfand AE, Smith AFM (1990). Sampling-based approaches to calculating marginal densities. J Am Stat Assoc.

[CR29] Gelman A, Roberts GO, Gilks WR (1996). Efficient metropolis jumping rules. Bayesian Stat.

[CR30] Gelman A, Carlin JB, Stern HS, Rubin DB (2003). Bayesian data analysis.

[CR31] Gilioli G, Pasquali S, Tramontini S, Riolo F (2013). Modelling local and long-distance dispersal of invasive chestnut gall wasp in europe. Ecol Model.

[CR32] Giunta A, Watson L (1998) A comparison of approximation modeling techniques-polynomial versus interpolating models. In: 7th AIAA/USAF/NASA/ISSMO symposium on multidisciplinary analysis and optimization, multidisciplinary analysis optimization conferences, St. Louis, MO, USA, p 4758. 10.2514/MMAO98

[CR33] Godefroid M, Cruaud A, Streito JC, Rasplus JY, Rossi JP (2018) Climate change and the potential distribution of *Xylella fastidiosa* in Europe. bioRxiv 10.1101/289876

[CR34] Gonze D, Halloy J, Goldbeter A (2002). Deterministic versus stochastic models for circadian rhythms. J Biol Phys.

[CR35] Hecht F (2012). New development in Freefem++. J Numer Math.

[CR36] Hengeveld R (1989). Dynamics of biological invasions.

[CR37] Henneberger TS (2003) Effects of low temperature on populations of *Xylella fastidiosa* in sycamore. Ph.D. thesis, University of Georgia

[CR38] Higgins SI, Richardson DM (1999). Predicting plant migration rates in a changing world: the role of long-distance dispersal. Am Nat.

[CR39] Huld TA, Šúri M, Dunlop ED, Micale F (2006). Estimating average daytime and daily temperature profiles within Europe. Environ Model Softw.

[CR40] Kareiva P, Shigesada N (1983). Analyzing insect movement as a correlated random walk. Oecologia.

[CR41] Kermack WO, McKendrick AG (1927). A contribution to the mathematical theory of epidemics. R Soc.

[CR42] Lanzarone E, Pasquali S, Gilioli G, Marchesini E (2017). A Bayesian estimation approach for the mortality in a stage-structured demographic model. J Math Biol.

[CR43] Lewis MA, Kareiva P (1993). Allee dynamics and the spread of invading organisms. Theor Popul Biol.

[CR44] Lindley D (2006). Understanding uncertainty.

[CR45] Martinetti D, Soubeyrand S (2018) Identifying lookouts for epidemio-surveillance: application to the emergence of *Xylella fastidiosa* in France, submitted 10.1094/PHYTO-07-18-0237-FI 30457431

[CR46] Mason SJ (2004) On using “climatology” as a reference strategy in the brier and ranked probability skill scores. Mon Weather Rev 132:1891–1895. 10.1175/1520-0493(2004)132<1891:OUCAAR>2.0.CO;2

[CR47] Mollison D (1977). Spatial contact models for ecological and epidemic spread. J R Stat Soc Ser B (Methodol).

[CR48] Murray JD (2002) Mathematical biology. In: Interdisciplinary applied mathematics, vol 17, 3rd edn. Springer, New York

[CR49] Nathan R, Schurr FM, Spiegel O, Steinitz O, Trakhtenbrot A, Tsoar A (2008). Mechanisms of long-distance seed dispersal. Trends Ecol Evol.

[CR50] Okubo A (1980). Diffusion and ecological problems: mathematical models, interdisciplinary applied mathematics.

[CR51] Okubo A, Levin S (2002). Diffusion and ecological problems—modern perspectives.

[CR52] Osio IG, Amon CH (1996). An engineering design methodology with multistage Bayesian surrogates and optimal sampling. Res Eng Des.

[CR53] Peterson RO, Vucetich JA, Page RE, Chouinard A (2003). Temporal and spatial aspects of predator–prey dynamics. Alces.

[CR54] Protter MH, Weinberger HF (1967). Maximum principles in differential equations.

[CR55] Purcell A (1977). Cold therapy of pierce’s disease of grapevines. Plant Dis Rep.

[CR56] Purcell A (1980). Environmental therapy for pierce’s disease of grapevines. Plant Dis.

[CR57] Reise K, Olenin S, Thieltges DW (2006). Are aliens threatening european aquatic coastal ecosystems?. Helgol Mar Res.

[CR58] Renshaw E (1993). Modelling biological populations in space and time.

[CR59] Richardson DM, Bond WJ (1991). Determinants of plant distribution: evidence from pine invasions. Am Nat.

[CR60] Roberts GO, Gelman A, Gilks WR (1997). Weak convergence and optimal scaling of random walk metropolis algorithms. Ann Appl Probab.

[CR61] Roques L, Soubeyrand S, Rousselet J (2011). A statistical-reaction–diffusion approach for analyzing expansion processes. J Theor Biol.

[CR62] Roques L, Walker E, Franck P, Soubeyrand S, Klein E (2016). Using genetic data to estimate diffusion rates in heterogeneous landscapes. J Math Biol.

[CR63] Schwarz G (1978). Estimating the dimension of a model. Ann Stat.

[CR64] Shigesada N, Kawasaki K (1997). Biological invasions: theory and practice.

[CR65] Shigesada N, Kawasaki K, Takeda Y (1995). Modeling stratified diffusion in biological invasions. Am Nat.

[CR66] Simberloff D (1989). Which insect introductions succeed and which fail?.

[CR67] Simpson TW, Poplinski J, Koch PN, Allen JK (2001). Metamodels for computer-based engineering design: survey and recommendations. Eng Comput.

[CR68] Skellam JG (1951). Random dispersal in theoretical populations. Biometrika.

[CR69] Soubeyrand S, Roques L (2014). Parameter estimation for reaction-diffusion models of biological invasions. Popul Ecol.

[CR70] Soubeyrand S, Laine AL, Hanski I, Penttinen A (2009). Spatio-temporal structure of host-pathogen interactions in a metapopulation. Am Nat.

[CR71] Soubeyrand S, Neuvonen S, Penttinen A (2009). Mechanical-statistical modeling in ecology: from outbreak detections to pest dynamics. Bull Math Biol.

[CR72] Soubeyrand S, de Jerphanion P, Martin O, Saussac M, Manceau C, Hendrikx P, Lannou C (2018). What dynamics underly temporal observations? Application to the emergence of *Xylella fastidiosa* in France: probably not a recent story. New Phytol.

[CR73] Spiegelhalter DJ, Best NG, Carlin BP, Van Der Linde A (2002). Bayesian measures of model complexity and fit. J R Stat Soc Ser B (Stat Methodol).

[CR74] Strona G, Carstens CJ, Beck PS (2017). Network analysis reveals why *Xylella fastidiosa* will persist in Europe. Sci Rep.

[CR75] Turchin P (1998). Quantitative analysis of movement: measuring and modeling population redistribution in plants and animals.

[CR76] Verhulst PF (1838) Notice sur la loi que la population suit dans son accroissement. In: Mathématique & sciences humaines, vol 167, Quetelet, pp 51–81

[CR77] Vermeij GJ (1996). An agenda for invasion biology. Biol Conserv.

[CR78] Weinberger H, Chadam JM (1978). Asymptotic behavior of a model in population genetics. Nonlinear partial differential equations and applications.

[CR79] White SM, Bullock JM, Hooftman DAP, Chapman DS (2017). Modelling the spread and control of *Xylella fastidiosa* in the early stages of invasion in Apulia, Italy. Biol Invasions.

[CR80] Wikle CK (2003). Hierarchical Bayesian models for predicting the spread of ecological processes. Ecology.

[CR81] Wikle CK (2003). Hierarchical models in environmental science. Int Stat Rev.

