# Supplementary material for: Dating and localizing an invasion from post-introduction data and a coupled reaction–diffusion–absorption model
Source: J Math Biol. 2019 May 16;79(2):765–89. doi: 10.1007/s00285-019-01376-x (PMC6647151; doi:10.1007/s00285-019-01376-x)
Supplement: Supplementary file 1 — Supplementary material 1 (pdf 5321 KB) [file 285_2019_1376_MOESM1_ESM.pdf]

Electronic Supplementary Material (EMS)

Dating and localizing an invasion from post-introduction data and a coupled reaction-diffusion-absorption model

C. Abboud[1], O. Bonnefon[1], E. Parent[2], and S. Soubeyrand[1]

[1]BioSP, INRA, 84914 Avignon, France

[2] AgroParisTech, UMR 518 Math. Info. Appli., Paris, France,

INRA, UMR 518 Math. Info. Appli., Paris, France

S1 Numerical Equation Solving

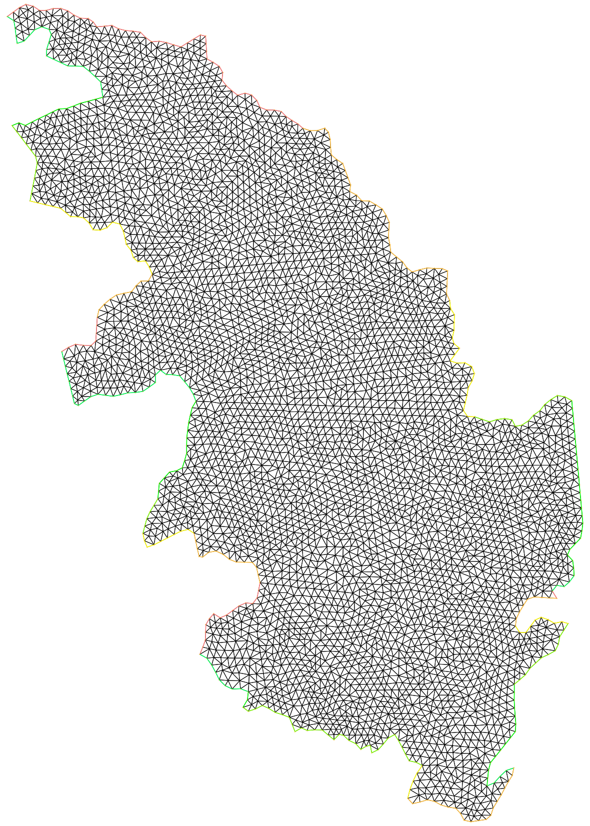

Figure S1: Mesh used for the Finite Element Method. This mesh contains 4791 nodes and 9141 triangles. The geometrical characteristics of this mesh were used to compute the accuracy of the simulator.

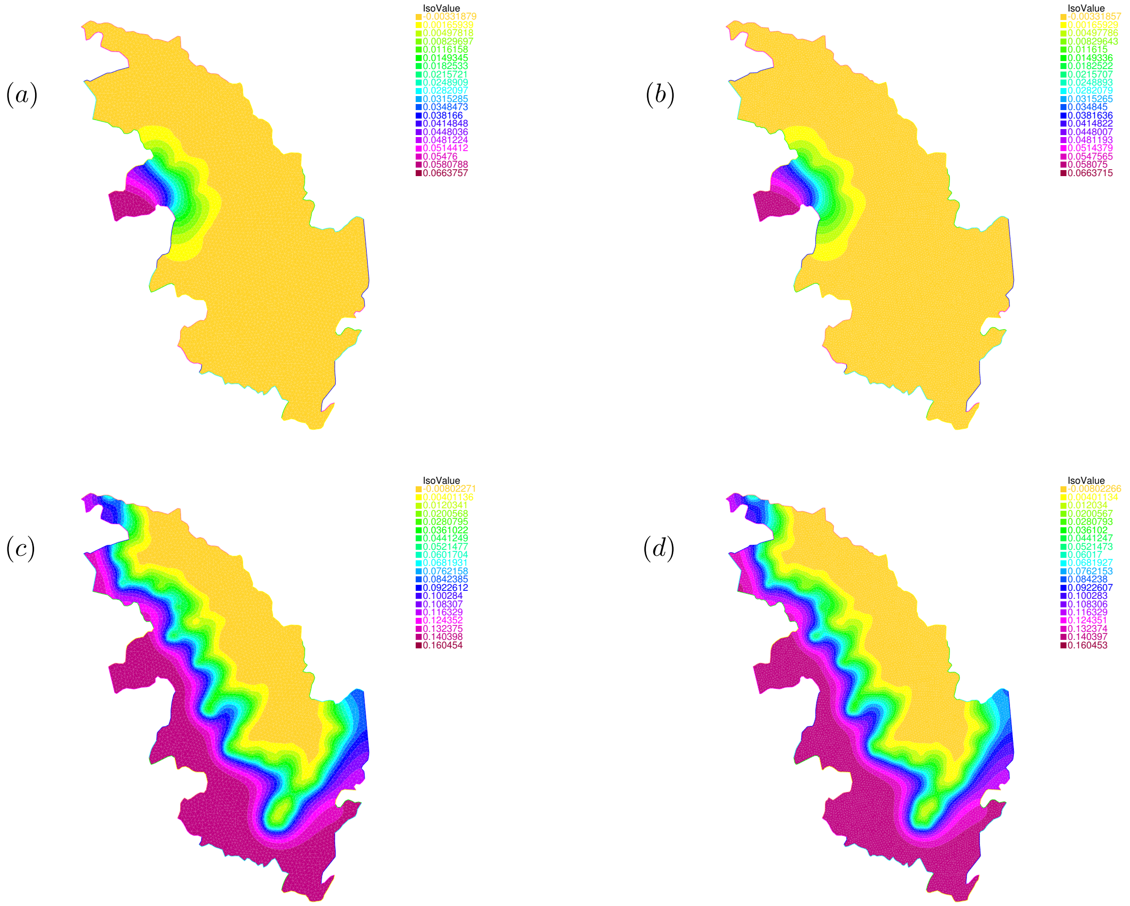

Figure S2: Probability of infection obtained at two different times and with two different meshes for the parameter vector corresponding to the posterior maximum. Top panels: 100 months after the introduction; Bottom panels: time of the last observation; Left panels: mesh composed of 4791 nodes; Right panel: finer mesh with 10703 nodes. Average difference between (a) and (b): $3e^{-5}$; Maximal difference: 0.002. Average difference between (c) and (d): $4e^{-5}$; Maximal difference: 0.02.

S2 Local Brier Score

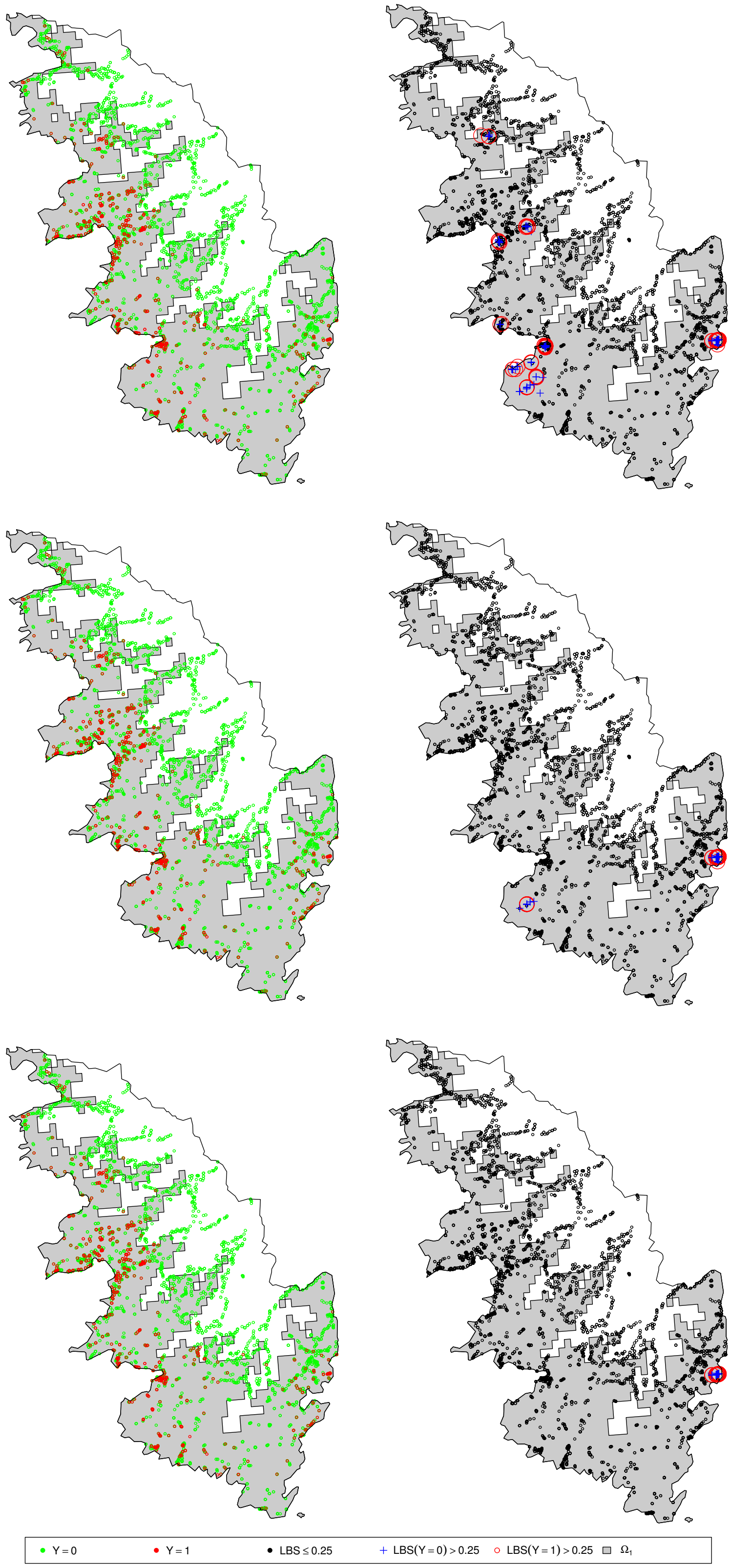

Figure S3: Locations where the LBS given in section 3.5.1 (main text) is larger than 0.25 with $k = 50$ (top), $k = 100$ (center), $k = 150$ (bottom). The gray surface gives the extent of Ω_1.

S3 Maximum likelihood estimation

Estimation of the parameter vector $\Theta = (D, b, K, \alpha, \tau_0, \tilde{\mathbf{x}}_{\mathbf{0}}, r_0, p_0)$ was also performed in the frequentist setting via maximum likelihood estimation. The maximization of the likelihood was made with the function `fmincon` of `Matlab R2015b`. This function searches for the optimum of a constrained nonlinear multivariable function using the Broyden-Fletcher-Goldfarb-Shanno (BFGS) algorithm. To take into account the risk of finding a local optimum, we carried out the maximization of the likelihood for 240 different initial values of the parameter vector drawn from the prior distribution. Figure S4 shows the evolution of the log-likelihood function from the initial values to the optimal values for the 240 calls of `fmincon`. We clearly see that most of the calls led to a relatively high likelihood (despite a few exceptions), but none of them led to a higher value than the highest value obtained with AMIS, which is not designed as an optimizer but is designed as a sampler in the posterior distribution (the highest log-likelihood value obtained with `fmincon` is -2467.8, whereas it is -2449.9 with AMIS). From a computational perspective, the maximum likelihood approach required 273 likelihood evaluations in average (i.e., 6.5×10^4 evaluations for the 240 optimizations), whereas we made 50×10^4 likelihood evaluations in AMIS. Thus, maximum likelihood estimation is less demanding, but is apparently stuck in local optimum with our model and data, and an additional task would be required to assess uncertainty in parameter estimation (e.g., via the computation of the Fisher information matrix), whereas AMIS directly provides estimation uncertainty.

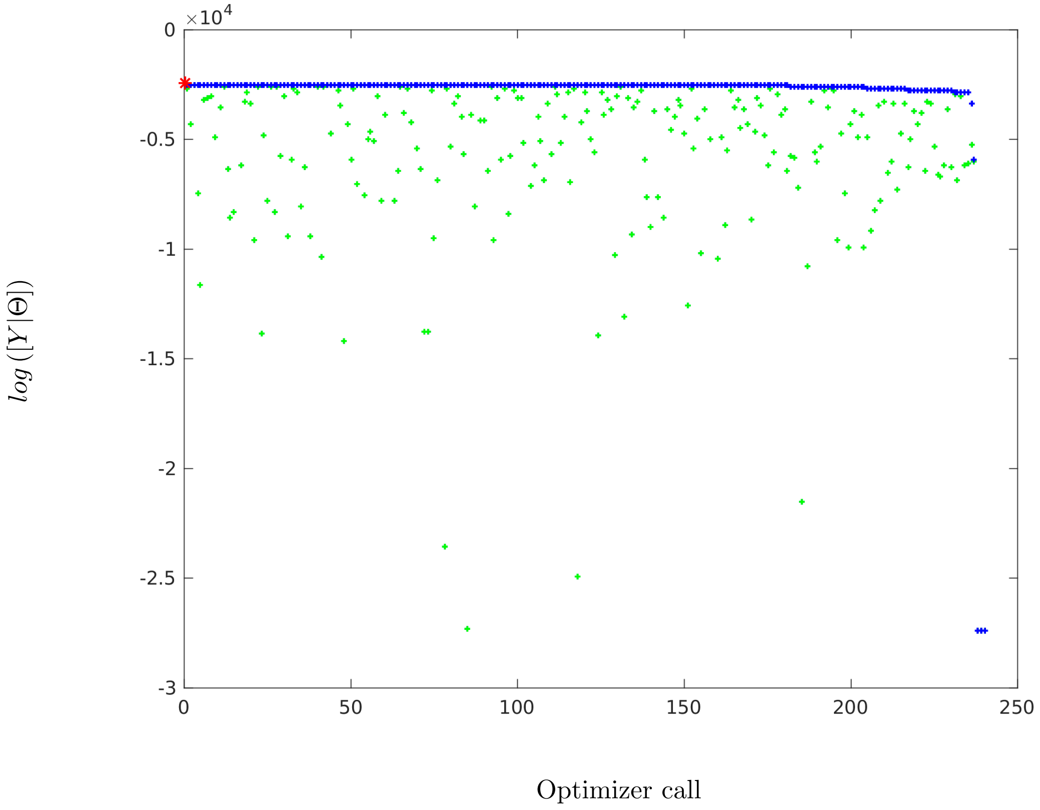

Figure S4: Values of the log-likelihood function evaluated at 240 different initial values of Θ used for the optimization (green crosses), and at the 240 resulting optimized values of Θ obtained with the fmincon function (blue crosses). The red asterisk indicates the maximum value of the log-likelihood obtained in the AMIS procedure.
